# Evaluation of bone healing using rhBMP-2 soaked hydroxyapatite in ridge augmentation: a prospective observational study

**DOI:** 10.1186/s40902-017-0138-9

**Published:** 2017-12-25

**Authors:** Hyun-Suk Kim, Ju-Cheol Park, Pil-Young Yun, Young-Kyun Kim

**Affiliations:** 1 Department of Oral and Maxillofacial Surgery, Section of Dentistry, Seoul National University Bundang Hospital, 300 Gumi-dong, Bundang-gu, Seongnam, Gyunggi-do South Korea; 2 Department of Oral Histology, School of Dentistry, Seoul National University, Daehak-ro 101, Jongno-gu, Seoul, 03080 South Korea; 3 Department of Dentistry and Dental Research Institute, School of Dentistry, Seoul National University, Daehak-ro 101, Jongno-gu, Seoul, 03080 South Korea

**Keywords:** Alveolar ridge augmentation, Bone morphogenetic protein 2, Bone regeneration, Hydroxyapatite

## Abstract

**Background:**

The goal of this study is to evaluate complication and effectiveness of alveolar ridge augmentations using a hydroxyapatite-based alloplastic bony substitute with rhBMP-2.

**Methods:**

A total of 10 patients (4 males, 6 females; 58.5 ± 8.6 years) participated in this clinical research. Alveolar ridge augmentations were performed in edentulous (4 maxillary posterior, 5 mandibular posterior, and 1 mandibular anterior) regions. Anorganic bovine bone (ABB; Bio-Oss®, Geistlich Pharma AG, Wolhusen, Switzerland) was used as the bone graft material in the control group (*n* = 5)) while hydroxyapatite-based alloplastic bony substitute with rhBMP-2(HA+rhBMP-2; NOVOSIS®-Dent, CGBio Inc., Seongnam, Korea) was used in the experimental group (*n* = 5). In order to evaluate relative changes in bone volume and resorption rate of the bone graft material, CBCT radiographs were taken immediately and at 4 months after the bone graft in all subjects. Among the 10 patients, 8 received dental implants in Seoul National University Bundang Hospital, while the others received in local clinics. Bone specimens for further histomorphometric examinations were gained from these 8 patients using trephine burs during the implant placements. Clinical, radiographic, and histomorphometric evaluations were focused because of the small sample size.

**Results:**

When CBCT radiographs were compared between immediately and at 4.07 ± 0.13 months after the bone graft, both alveolar bone widths (ABB 2.52 ± 0.18 mm, HA+rhBMP-2 1.75 *±* 0.85 mm) and heights (ABB 1.68 ± 0.17 mm, HA+rhBMP-2 1.57 ± 0.28 mm) increased in the two groups. Resorption rates of transplanted bone graft material in the alveolar bone widths and heights were (ABB 29.7 ± 8.8%, HA+rhBMP-2 31.5 ± 7.4%) and (ABB 39.2 ± 21.8%, HA+rhBMP-2 52.6 ± 6.5%), respectively. Histomorphometrically, ABB group showed bone formation via osteoconduction and HA+rhBMP-2 group via osteoinduction. HA+rhBMP-2 group showed more bone formation around the bone graft materials than the ABB group. Postoperative complications were not found in all subjects.

**Conclusions:**

Our study had following conclusions: (1) Ridge augmentations using HA+rhBMP-2 could be clinically useful to supplement implant placements in edentulous regions. (2) Serious postoperative complications related to the graft material did not occur.

**Electronic supplementary material:**

The online version of this article (10.1186/s40902-017-0138-9) contains supplementary material, which is available to authorized users.

## Background

Adequate bone volume is one of the important factors to obtain osseointegration in dental implants. In 1986, Lekholm et al. reported that for the implant success, a minimum of 1 mm or more of the buccal and lingual bone was necessary surrounding the implant surface. Clinicians often encounter patients with deficient vertical or horizontal alveolar bones. Reasons may vary from trauma, periodontal disease, tooth extraction, and to tumor. Under such circumstances, the implant surface may not be entirely covered by the bone, and this could increase the risk of infection, gingival recession, non-esthetic appearance, poor oral hygiene maintenance, and peri-implantitis [1].

The ideal bone graft material should have no immune response and include growth factors that facilitate rapid bone formation and re-vascularization. It should also be able to maintain space for new bone infiltration and readily available in clinics.

Autografts are known to be the ideal material for the reconstruction of bone defects. Autografts have osteogenesis, osteoconduction, and osteoinduction abilities that enable rapid bone healing without inducting immune responses. They are, however, difficult to obtain in sufficient quantities without causing complications in the donor site and a large amount of the transplanted grafts often get absorbed. To overcome the problems, other bone substitutes such as allografts, xenografts, and alloplasts have been developed and used. However, allografts and xenografts could be problematic due to the risk of infection and high price. The alloplasts are cheap and have no risk of infection, but they lack osteogenesis and osteoinduction abilities to form viable bone tissue [2].

Bone morphogenetic protein (BMP), the leading osteoinductive growth factor, has been studied since 1995 and extensively in the 2000s. Animal studies focused on discovering roles of BMP in guided bone regeneration (GBR) when delivered with drug carriers [3, 4]. In general, osteoinductive capabilities should be given to osteoconductive bone graft materials for bone graft material development. Various proteins, such as BMP-2, 4, 7, and 14, have been reported to have osteoinductive abilities in animal experiments.

In particular, BMP-2 act as a growth and differentiation factor in the body and promotes the new bone formation by acting extensively at the entire stage of osteogenesis ranging from mesenchymal stem cells-osteoprogenitor-preosteoblast-osteoblast-osteocytic osteoblast-osteocyte [5].

BMP-2 also showed potential for bone regeneration through various studies including sinus augmentation [6, 7], alveolar bone preservation [8], bone augmentation [9], and periodontal recovery [10]. Ike and Urist reported that BMP-2 contained in dentin exhibited osteoinductive abilities important for osteogenesis [11]. Jung et al. showed that GBR with combination of the xenograft (Bio-Oss) with rhBMP-2 can enhance the maturation process of bone regeneration [12].

Similar to other growth factors, BMP-2 requires a carrier system that could provide optimal cellular and vascular growth, cellular attachment, and release kinetics [13, 14]. Highly soluble, BMP-2 requires robust scaffolds over long periods that act as drug carrier at the implant site to exert osteoinductive effects [15, 16]. Ideal scaffolds should control-release growth factors and prevent degradations. Various materials have been proposed, and absorbable collagen sponge (ACS) has been the most documented carrier for rhBMP-2 because of its high binding and retention capacities for the rhBMP-2. According to a study by Hwang, the use of rhBMP-2 with ACS could result in accelerated bone formation compared to conventional bone grafting in postoperative bone defects [17]. However, collagen lacks osteoconductivity as well as structural integrity in transplanted sites. Therefore, calcium phosphates, such as hydroxyapatite (HA) and β-tricalcium phosphate (β-TCP), have been considered as suitable candidates for rhBMP-2 delivery system because of their space-providing properties [18].

The goal of this study is to evaluate effectiveness and complication of alveolar ridge augmentations using a hydroxyapatite-based alloplastic bony substitute with rhBMP-2.

## Methods

### Patients

A total of 10 patients (4 males, 6 females; 58.5 ± 8.6 years) participated in this clinical research. Alveolar ridge augmentations were performed in edentulous (4 maxillary posterior, 5 mandibular posterior, and 1 mandibular anterior) regions.

### Surgical procedure

All patients received alveolar bone augmentation in the deficient ridge areas. Under local anesthesia using 1% lidocaine with 1:100,000 epinephrine (Huons, Hwasung, Korea), vertical and horizontal incisions were made in the mucoperiosteum of the labial or buccal sides of edentulous regions. A periosteal flap was elevated with a periosteal elevator, and a selection of bone graft materials was placed underneath the highly cross-linked resorbable collagen membrane (Ossix Plus, Datum Dental Ltd., Telrad, Israel). Anorganic bovine bone (ABB; Bio-Oss®, Geistlich Pharma AG, Wolhusen, Switzerland) was used as the bone graft material in the control group (*n* = 5) while hydroxyapatite-based alloplastic bony substitute with rhBMP-2(HA+rhBMP-2; NOVOSIS®-Dent, CGBio Inc., Seongnam, Korea) was used in the experimental group (*n* = 5). NOVOSIS®-DENT uses synthetic grafting bone (hydroxyapatite) as multi-pore ceramic supporter to convey BMP-2 to the human body. Graft materials were prepared according to manufacturer’s instructions. The mucoperiosteal flaps were then closed with 4–0 Vicryl (polyglactin; Ethicon Inc., Sommerville, NJ) using a simple interrupted suture technique.

Patients who underwent surgery took antibiotics (amoxicillin/clavulanate; Augmentin®, Ilsung Pharmaceuticals Co., Seoul, Korea) and a non-steroidal anti-inflammatory drug (talniflumate; Somalgen®, Kunwha Pharmaceutical Co., Seoul, Korea) for 5 days postoperatively. A 100 mL of 0.1% chlorhexidine mouth gargling (Hexamedine®, Bukwang Pharm, Ansan, Korea) was prescribed for oral hygiene maintenance. Sutures were stitched out between 1 and 2 weeks after the surgery.

### Case

**Fig. 1:**
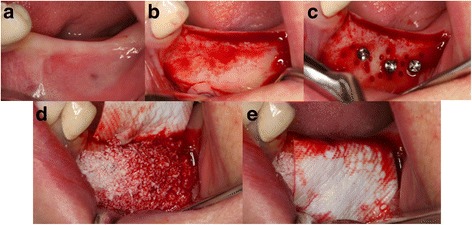
Intraoral photographs of ridge augmentation using bone graft material and resorbable collagen membrane. **a** Deficient alveolar ridge. **b** Mucoperiosteal flap elevation. **c** Tenting screws installed around the bone grafts. **d** Bone graft placed. **e** Resorbable collagen membrane applied

A 60-year-old female came to our department for restoration of left mandibular premolar and molar areas. On deficient alveolar ridge, ABB with resorbable collagen membrane was grafted. To radiographically evaluate the bone resorption and formation, as well as to provide support for graft materials, tenting screws were installed around the bone grafts (Fig. 1).

### Measurement of alveolar bone volume change

**Fig. 2:**
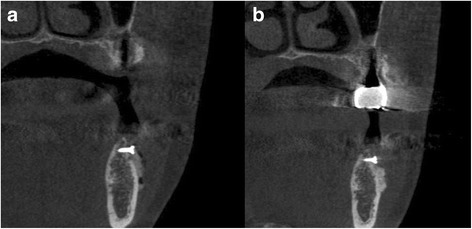
CBCT, postoperative view. **a** Immediately after the bone graft. **b** Four months after the graft

To evaluate relative changes in bone volume and resorption rate of the bone graft material, three dimensional measurements were obtained by cone-beam computed tomography (CBCT) immediately and at 4 months after the bone graft in all subjects (Fig. 2). The changes in bone width and height between the two groups were calculated, and Mann-Whitney *U* test (SPSS Inc., Chicago, IL, USA) was used to evaluate statistical significance. *P* values less than 0.05 were considered to be statistically significant.

Among 10 patients, 8 received dental implants in Seoul National University Bundang Hospital, while the others received in local clinics.

### Histomorphometric assessment

Bone specimens for further histomorphometric examinations were gained from the 8 patients who received implants in Seoul National University Bundang Hospital (control *n* = 4, experimental *n* = 4) using trephine burs during the implant placements. The acquired specimens were decalcified using 10% formic acid for 3 weeks, embedded in paraffin, and sagittal sections were obtained and then stained with hematoxylin and eosin (H&E) for histologic examinations. Because of a small sample size, histologic, radiographic, and clinical evaluations were focused. To determine the relative amount of bone formation, the new bone formation ratio was measured using the analySIS LS starter program. The new bone formation was observed around the material in each of the 4 patient samples of each selected group, and the mean values of the areas were calculated. Statistical difference between bone formation of the two groups were calculated using Mann-Whitney *U* test.

This study was approved by the Institutional Review Board of Seoul National University Bundang Hospital (E-1501-282-001).

## Results

Postoperative complications were not found in all subjects.

### Augmented bone volumes and resorption rates

When CBCT radiographs were compared between immediately and at 4.07 ± 0.13 months after the bone graft, both alveolar bone widths (ABB 2.52 ± 0.18 mm, HA+rhBMP-2 1.75 *±* 0.85 mm) and heights (ABB 1.68 ± 0.17 mm, HA+rhBMP-2 1.57 ± 0.28 mm) increased in the two test groups. Resorption rates of transplanted bone graft material in the alveolar bone widths and heights were (ABB 29.7 ± 8.8%, HA+rhBMP-2 31.5 ± 7.4%) and (ABB 39.2 ± 21.8%, HA+rhBMP-2 52.6 ± 6.5%), respectively (Table 1). Significant differences were not found in bone width and height resorptions between ABB and HA+rhBMP-2 groups (width *p* = 0.841, height *p* = 0.548).

**Table 1:** Amount of bone augmentation

Group	Width			Height		
	T_1_ (mm)	T_4_ (mm)	Bone resorption (%)	T_1_ (mm)	T_4_ (mm)	Bone resorption (%)
ABB	3.7 ± 0.8	2.5 ± 0.2	29.7 ± 8.8	3.1 ± 1.4	1.7 ± 0.2	39.2 ± 21.8
HA+rhBMP-2	2.6 ± 1.2	1.8 ± 0.9	31.5 ± 7.4	3.3 ± 0.5	1.6 ± 0.3	52.6 ± 6.5
*P* value			0.841			0.548

Abbreviations: *ABB* anorganic bovine bone, *HA* hydroxyapatite, *rhBMP-2* recombinant human bone morphogenetic protein-2, *T*_*1*_ immediately postoperative augmented bone, *T*_*4*_ augmented bone after 4 months after the graft

### Histomorphometric findings of new bone formation

**Fig. 3:**
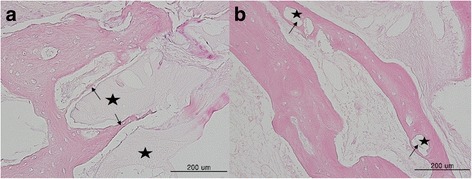
Histologic findings after 4 months of bone healing. **a** ABB. **b** HA+rhBMP-2. New bone formations (arrows) are observed between the graft materials (stars) and adjacent bones

**Fig. 4:**
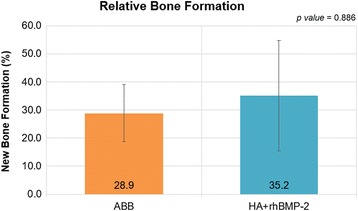
Relative bone formation. HA+rhBMP-2 group showed more bone formation around the bone graft materials than the ABB group histologically, however, without statistical significance (*p* = 0.886)

Bone formations were observed in both groups, but the appearance was different. Histomorphometrically, HA+rhBMP-2 group showed more bone formation around the bone graft materials than the ABB group (Fig. 3). Osteoconduction was observed around the material in the ABB group. In other words, ABB served as a bridge to bone formation as a scaffold. In the HA+rhBMP-2 group, osteoinduction occurred around the material and bone formation was observed. HA+rhBMP-2 group (35.2 ± 19.7%) showed more relative bone formation compared to the ABB group (28.9 ± 10.3%), but significant difference was not found (*p* = 0.886) (Fig. 4).

## Discussion

Despite possessing good biocompatibility and osteoconductive potential, most of commercially available graft materials lack osteoinductive potential. Much research thus has been focused on graft materials mixed with additives that could promote osteogenic potentials such as bone morphogenetic protein (BMP).

Kim et al. studies showed that demineralized dentin matrix (DDM) could act as an effective rhBMP-2 carrier [4, 13]. Kim also had reported that HA or DDM scaffolds could be combined with rhBMP-2 and promote bone formation [19].

A study from Kim et al. showed that low-dose *Escherichia coli*–derived rhBMP-2 with HA is as effective as anorganic bovine bone xenografts in early stages for enhanced bone formation after maxillary sinus floor augmentation without any major intraoperative or postoperative complications [7]. The soft tissue and residual graft areas showed no significant differences between the groups and rhBMP-2 antibody in the serum after BMP-2/H grafting did not increase significantly.

A study by Burkus et al. showed that formation of anti-BMP-2 antibodies are low and transient in patients treated with rhBMP-2 [20]. Moreover, small formation of antibodies did not affect fusion success and had no visible affect clinical sequelae.

Many studies support that BMP-2 is an effective osteoinducer, and there is no evidence that administration of rhBMP-2 at the time of surgery links with an increased risk of cancer [21, 22]. However, rhBMP-2 could induce adverse clinical effects, including ectopic bone formation and tissue inflammation when used in high concentrations [23, 24].

NOVOSIS®-Dent is a graft material used in combination with rhBMP-2 and HA carrier in alveolar bone defect areas. In order for rhBMP-2 to exert its effects, it must act locally at the site where new bone formation is required, and for this reason, it is commonly used with carriers capable of releasing from local sites. The carrier of NOVOSIS®-Dent is HA, a material that occupies 65% of the bone and 98% of the dental enamel, and it provides osteoconduction by providing a porous structure (83% porosity, 300 μm pore size). NOVOSIS®-Dent provides osteoconduction by using HA as a carrier and osteoinduction capability by utilizing BMP-2 to form a new bone at the bone defect site.

In this study, HA+rhBMP-2 showed relatively good bone formation compared with ABB. Although HA+rhBMP-2 had less volume of bone augmentation (widths ABB 2.52 ± 0.18 mm, HA+rhBMP-2 1.75 *±* 0.85 mm; heights ABB 1.68 ± 0.17 mm, HA+rhBMP-2 1.57 ± 0.28 mm) and more resorptions over 4-month periods (widths ABB 29.7 ± 8.8%, HA+rhBMP-2 31.5 ± 7.4%, *p* = 0.841 and heights ABB 39.2 ± 21.8%, HA+rhBMP-2 52.6 ± 6.5%, *p* = 0.548), it showed relatively more bone formation in histomorphometric assessments (ABB 28.9 ± 10.3%, HA+rhBMP-2 35.2 ± 19.7%; *p* = 0.886). This suggests that HA+rhBMP-2 group may provide new bone formation through osteoinductive abilities provided by the osteogenic protein. This characteristic observation might be consistent with the in vivo and in vitro studies that the delivered rhBMP-2-activated dentin resorption that was associated with giant cells, ultimately promoting the bone formation and remodeling capacity in later stage [13, 25]. However, even though the average bone formation was higher in HA+rhBMP-2 group, the statistical difference was marginal because of the limited number of the cases. Therefore, the result needs to be confirmed by the more number of the cases in the future study.

## Conclusions

Bone graft material including rhBMP-2 showed good bone formation and remodeling capabilities. Within its limitation, this study suggested that ridge augmentations using rhBMP-2 soaked HA could be clinically useful to supplement implant placements in edentulous regions. Additionally, serious postoperative complications related to the graft material did not occur.

## Additional file

Additional file 1: Case form and result of data. (XLSX 37 kb)
